# Mothers’ perception of the care of newborn in the home environment

**DOI:** 10.1590/0034-7167-2023-0080

**Published:** 2024-04-22

**Authors:** Gina de Souza Castro Hammel, Laura Tais Loureiro Simas, Luiz Fernando Rodrigues, Claudia Zamberlan, Lurdes Lomba, Dirce Stein Backes

**Affiliations:** I Universidade Franciscana. Santa Maria, Rio Grande do Sul, Brazil; II Escola Superior de Enfermagem. Coimbra, Portugal

**Keywords:** Nursing Research, Infant, Newborn, Mothers, Home Care Services, Child Care, Investigación en Enfermería, Recién Nacido, Madres, Servicios de Atención de Salud a Domicilio, Cuidado del Niño, Pesquisa em Enfermagem, Recém-Nascido, Mães, Serviços de Assistência Domiciliar, Cuidado da Criança

## Abstract

**Objectives:**

to identify mothers’ perceptions about caring for newborns in the home environment, from the perspective of complexity thinking.

**Methods:**

qualitative, exploratory and descriptive research, carried out between November/2022 and February/2023. Data were collected through individual interviews with 21 mothers from southern Brazil who cared for newborns at home and analyzed using the thematic analysis technique.

**Results:**

the four thematic axes resulting from the data analysis: Living amidst order and disorder; embracing singularities; dealing with the certain and the uncertain; support network in the (re)organizing process demonstrate that the mother caring for a newborn in their home environment experiences a distinct and plural adaptive process, which must be welcomed and understood by health professionals who work within the family environment.

**Final Considerations:**

the care of newborns in a home environment, in the perception of mothers, requires differentiated attention and a formal or informal support network that considers the unique specificities of each woman/mother in the personal, family and social spheres. Therefore, in addition to the social support network, it is important to rethink home intervention approaches.

## RESUMO

**Objetivos:**

identificar percepções de mães sobre o cuidado de recém-nascidos em ambiente domiciliar, na perspectiva do pensamento da complexidade.

**Métodos:**

pesquisa qualitativa, de caráter exploratório e descritivo, realizada entre novembro/2022 e fevereiro/2023. Os dados foram coletados por meio de entrevistas individuais com 21 mães do sul do Brasil que cuidaram de recém-nascidos em ambiente domiciliar e analisados pela técnica de análise temática.

**Resultados:**

os quatro eixos temáticos resultantes da análise dos dados: Convivendo em meio a ordem e desordem; acolhendo às singularidades; lidando entre o certo e o incerto; rede de apoio no processo (re)organizador demonstram, que a mãe cuidadora de um recém-nascido em ambiente domiciliar vivência um processo adaptativo distinto e plural, que deve ser acolhido e compreendido pelos profissionais de saúde que atuam em âmbito familiar.

**Considerações Finais:**

o cuidado de recém-nascidos em ambiente domiciliar, na percepção de mães, requer atenção diferenciada e uma rede de apoio formal ou informal que considere as especificidades singulares de cada mulher/mãe em âmbito pessoal, familiar e social. Assim, para além da rede de apoio social é importante que se repense as abordagens de intervenção domiciliar.

## RESUMEN

**Objetivos:**

identificar las percepciones de las madres sobre el cuidado de los recién nacidos en un entorno domiciliario, bajo la perspectiva del pensamiento de la complejidad.

**Métodos:**

es una investigación cualitativa, de carácter exploratorio y descriptivo, realizada entre noviembre de 2022 y febrero de 2023. Los datos se recopilaron mediante entrevistas individuales a 21 madres del sur de Brasil que cuidaron de neonatos en entorno domiciliario y se estudiaron bajo la óptica de la técnica del análisis temático.

**Resultados:**

los cuatro ejes temáticos resultantes del análisis de los datos fueron: convivencia en medio del orden y del desorden; acogimiento de las singularidades; lucha entre lo cierto y lo incierto; red de apoyo en el proceso de (re)organización. La madre que cuida de un recién nacido en entorno doméstico experimenta un proceso adaptativo distinto y plural, que debe ser atendido y comprendido por los profesionales sanitarios que trabajan en el ámbito familiar.

**Consideraciones Finales:**

el cuidado de los recién nacidos en un entorno doméstico, según la percepción de las madres, requiere una atención diferenciada y una red de apoyo formal o informal que tenga en cuenta las especificidades únicas de cada mujer/madre en los ámbitos personal, familiar y social. Por consiguiente, además de la red de apoyo social, es importante replantearse los enfoques de intervención en la atención domiciliaria.

## INTRODUCTION

In the pregnancy-puerperal cycle, pregnant women experience a period of instability and adaptations, often accompanied by disorder and (re)organization. This (re)organizing process intensifies postpartum, as there is a change of focus for the newborn, who demands essential care related to hygiene, breastfeeding, thermal maintenance and others, provided in most cases. of cases by the mother who, equally, needs specific support and care^(1)^.

In this sense, both prenatal care, childbirth and postpartum can be conceived as complex phenomena. In addition to physical and emotional changes, the pregnant/postpartum woman/ mother goes through a period of intense transformations that, in general, imply the (re)adaptation of routines in the personal, family, work and social spheres. Thus, the pregnancy-puerperal journey requires health professionals to take a singular and multidimensional approach, that is, woven by a set of threads/ factors that evoke, at least, more than one circumstance or interactive possibility to form integrated knowledge of the mother-baby binomial^(2,3)^.

The postpartum period is a complex period, especially for first-time mothers who need self-learning and specific care for their newborn. The lack of a support network, at this stage, represents a risk for the development of mental health problems^(4,5,6)^. A study shows that first-time mothers have unique emotional needs and that both formal and informal social support are important protective factors in the postpartum period^(7,8)^.

Although the support network has been mentioned as an important protective factor for the well-being of mothers caring for newborns at home, little is known about the social support needs of first-time mothers. Effective initiatives to promote the mother’s health also impact the health of the child and the family^(9)^. It is understood that providing a home support network for first-time mothers constituted, in this sense, an effective strategy in preventing favorable maternal and neonatal outcomes.

Other studies emphasize the relevance of providing tools and supporting parents in the care of newborns and children, with a view to reducing infant mortality rates and, in this way, contributing to achieving the Sustainable Development Goals^(10,11)^. Prioritizing improving the quality of care for newborns to reduce complications in the first days of life will certainly have an impact on reducing neonatal and infant mortality rates^(12,13)^.

Considering that postpartum home care is the best method to guarantee support for first-time mothers and with the aim of contributing to new thinking and acting in relation to the experiences of mothers caring for newborns, the present study aims to research question: what is the perception of mothers about the care of newborns in the home environment, from the perspective of complex thinking?

Complex thinking leads to a broader and contextualized view of the different phenomena that encompass health care. It also understands and conceives the order/disorder/organization relationship as necessary, under certain conditions, for the production of (re)organizing phenomena that contribute to increasing order and the evolution of knowledge in specific areas. Complexity does not refer to the number of components in a system, but to the richness of their relationships and associations^(14)^.

## OBJECTIVES

To identify mothers’ perceptions about caring for newborns in the home environment, from the perspective of complex thinking.

## METHODS

### Ethical aspects

In the research process, the recommendations of National Health Council Resolutions No. 466/2012 and the recommendations of Circular Letter No. 2/2021, referring to online research, were considered. The research project was approved by the Research Ethics Committee and written informed consents were obtained from all individuals involved in the study. To maintain anonymity, the participants’ statements were identified, throughout the text, with the letter ‘M’, for Mother, followed by a number corresponding to the order of the statements: M1...M21.

### Theoretical-methodological framework

The assumptions of complex thinking were used as a theoretical framework, which conceives the singularity and complexity of existential and health care phenomena. This thinking favors an expanded and systemic understanding of different social objects and leads to the search for new possibilities to intervene in the dynamics of caring for newborns in the home environment. This leads to a unique, circular and interactive process, driven by diverse experiences, in which the mother figures as the main protagonist^(14)^. The aim is a methodological path in which the researcher is induced to learn and (re)invent himself along the way, through autonomous and evolution-inducing processes both in the way of thinking and evolving and in the (re)innovation of care practices^(15)^.

### Study Design

This is qualitative research, of an exploratory and descriptive nature. Its approach, in light of complexity thinking, aims for meanings that expand perceptions, perspectives and experiences that cannot be reduced to linear variables. To this end, this investigation followed the Consolidation Criteria for Qualitative Research Reports (COREQ from the Portuguese – its original language).

### Methodological procedures

#### Study Setting

The corpus of this study was composed of 21 mothers living in southern Brazil, who cared for low-risk newborns in a home environment. Among the interviews, 16 were carried out in person, in the mother’s own home, and five were carried out online, depending on the geographic distance of the participants. The Google Meet platform was adopted to conduct online interviews. They were recorded and subsequently transcribed for analysis.

#### Participants selection

The participants were accessed for convenience, via the researchers’ personal Instagram and, after expressing their support for participation, they were contacted by email, in person, for further clarification and to schedule a day and time for data collection. The study included mothers caring for low-risk newborns (up to 28 days) in the home environment. Mothers under 18 years old were excluded from the study.

#### Dada collection and organization

Data collection took place between November/2022 and February/2023, through individual interviews, lasting an average of 40 minutes. The interviews were previously scheduled and conducted by an experienced researcher, based on guiding questions, which were deepened throughout the investigation: What was it like for you (mother) to care for the newborn at home? In your opinion, what is relevant and should be considered when caring for newborns at home? What is your suggestion for improving newborn care at home? Along the way, the main researcher organized a record file with the raw data investigated and, systematically, carried out the immersion through floating reading and the allocation of codes in a table, in order to identify the regularity of the findings and note the repetition of words. Thus, the saturation point was reached when no new elements were found and the addition of new information was no longer necessary to understand the phenomenon under investigation.

#### Data analysis

The data was coded based on the Reflexive Thematic Analysis technique, in order to enable the systematic recording of ideas and insights, in addition to providing a fluid and flexible coding of the meanings attributed by the participants. In this journey, we sought not only to achieve accuracy, but also deep and meaningful immersion in the data, in light of complexity thinking. To this end, the six phases of Thematic Analysis were followed: Familiarization with the data, based on repeated readings and a draft list of ideas; Generation of initial codes, manually, by systematizing relevant extracts; Search for themes based on the classification of different codes; Refinement of themes based on validation of initial themes; Naming the themes based on the essence that each theme portrays in its set of codes; and the Production of the report that will offer a reflective and prospective description of the investigation process as a whole^(16)^.

Reflexive Thematic Analysis sought, from obtaining descriptive data from the perspective of interpretative research, to signify mothers’ perception of the care of newborns in a home environment, in light of complexity thinking. In addition to the objectification of meanings, we sought the representativeness of each speech in the group of speeches. In this way, only the most significant speeches are presented in the totality of the data collected.

The process of analysis and meaning of the data resulted in four thematic axes, namely: Living amidst order and disorder; Embracing singularities; Dealing with the certain and the uncertain; Support network in the (re)organizing process.

## RESULTS

The participants in this study, 21 mothers who cared for low-risk newborns in a home environment, were on average 24 years old, had two children and, for the most part, had completed primary education and had an income between two and three minimum wages.

### Living amidst order and disorder

Pregnant women, in general, conceive a certain order in the prenatal period, which is profoundly altered postpartum, both in the personal and family spheres, at work and in social life. Indescribable feelings, expectations and emotions alternate. For self-employed mothers who need to return to work early, there is the feeling and discomfort of not fulfilling their duties.

*Returning home is always a unique, unforgettable and difficult moment to adapt to. Everything is new, everything changes. But it’s something inexplicable, which often makes you want to run away, but then you want to come back.* (M2)

*It’s a lot of change. Being a mother, combined with female demands, creates an immense change in a woman’s life*. (M6)

*Absolutely everything was very difficult. I’m self-employed, I had to go back to work early and that was my biggest difficulty, balancing my work and caring for* [son’s name] *at home. I often feel that I am not carrying out motherhood or my work as I would like. I feel like I’m not fully in any role*. (M11)

Most mothers experience intense emotional and sleep disorder. In many cases, the few hours of sleep at night were associated with inexperience and, in other cases, with the lack of an effective support network. One mother, in particular, reported exhaustion and lack of patience in dealing with her child’s demands and recognizes that these feelings may have impacted/difficulty forming a bond with her child.

*We sleep a maximum of two hours. She has had a lot of colic since she was born. It seems crazy, but we even woke up to check if she was breathing.* (M3)

*I remember that irregular sleep was a very complicated factor, because being alone I didn’t rest. This only got better when my husband’s grandmother arrived to help me. It was a big challenge. I had no experience. Even though I have read and studied about baby care, when I had to take care of him I went through insecure situations, especially in relation to bathing and breastfeeding*. (M14)

*If I had prepared myself better about the issue of sleep deprivation, the adaptation would have been a little smoother. Going without sleep for consecutive nights caused great discomfort and even hindered the formation of a bond with my son. I was always exhausted and had no patience to deal with all the demands he needed.* (M21)

Some mothers reported significant disorders associated with the dynamics of feedings, cracks in the breasts, milk let-down, latching on to the breast, frequency of feedings and others. These difficulties were more pronounced in mothers who had a C-section. Other mothers also highlighted (re)adaptations associated with the “older brother” routine.

*It was very difficult in the first few days due to the post-surgery period. And having to breastfeed was even more difficult.* (M4)

*It was difficult. The nipple was cracked and the baby was hungry, sucking all the time and only sleeping for a few hours. Everything was very difficult*. (M9)

*Breastfeeding was not easy at all despite us having studied and studied a lot on the subject. The frequency of feedings left me exhausted. But little by little he and I adapted. We also had to adapt our routine according to our older brother. Everything was new for him too*. (M19)

Difficulties related to bathing the baby were also expressed by several mothers. The fact that she was alone, that she didn’t know how to position him for the bath and the fear of hurting him were some of the reports presented.

*Everything was very difficult. The main memory I have was the difficulty with bathing, how to pick up the baby without hurting him. So, it was a very difficult period of sleep deprivation and baby care. Seriously, I don’t know how people have more than one child. Going through this more than once is crazy.* (M16)

*The whole adaptation was very difficult. But what bothered me most was the baby’s bath. I didn’t feel safe taking a bath alone and I had no one to help me.* (P20)

It was noticed, in almost all statements, that caring for newborns in a home environment is a highly complex process. Although many mothers have sought qualifications during the gestational period, the experience of being a mother and providing care for a newborn child involves a mix of feelings and disorders. Therefore, there is no ready-made recipe to equip mothers in relation to the care of the newborn, but it is possible to think of strategies that qualify prenatal care with clear and objective guidelines and that favor the postpartum period with more security and empowerment.

### Embracing singularities

It was noted, in the mothers’ speech, that there are no specific and linear protocols in relation to the care of newborns in the home environment. They mentioned that each child is unique and that, equally, they require unique care that is not found in books, even if some are “first, second, third time” mothers. Therefore, each child must be welcomed and cared for in their uniqueness, as expressed:

*It’s a unique experience. We always try to learn about essential care, but each child is unique. There is no single standard of care, she needs love, food and lap, right. Oh, having talked about the baby’s arrival with my older sister helped a lot. She is three years old. I talked to her a lot and it helped me not to be jealous, you know. She included the baby in everything and in her own way.* (M5)

*With my third child, everything was calmer. Now he is five months old. Things have improved a lot. The three are very different. Each one is very different.* (M7)

*Because even as a mother for the first, second, third time, each child is unique, right? Everything is very different from one to the other.* (M8)

*As a first-time mother it was a great challenge, but a unique learning experience that I didn’t find in books.* (M17)

Mothers who have more than one child recognize that each child has their own particularities and “what works for one does not necessarily work for the other”. They also recognize that mothers need to be equipped to provide differentiated and unique care, in addition to protocols and guidance manuals.

*We must always study, right, find out more and more because babies are very different. What works for my baby doesn’t necessarily work for my colleague’s baby. Each person adapts according to their condition and support network.* (M15)

*We already have another 6-year-old son. In a way, it helped a lot because we had already gone through that crazy part of the postpartum period. Even though we know that each child is unique and has their own particularities and differences, the routine took a general turnaround at home.* (M19)

Other mothers reported that they experienced each day intensely and that they tried to make sense of their daily experiences and learning. They consider it relevant, in this process, to promote aggregating and private environments, especially in the first weeks of the child’s life, in order to favor the necessary adaptations and personal and family (re)organizations.

*As we had been through this before, it was easier, even though the care is very different from one to the other. Let’s live one day at a time, because each one is unique and has its own particularities. Every day is a new learning.* (M1)

*The main thing is to maintain the privacy of parents and baby as soon as they arrive home. They need to have an adaptation period for everyone.* (P18)

*I was always the one who took care of him until he was 4 months old. It was a unique experience. I breastfed until I was two months old, I woke up several times during the night and after that I started using formula and we slept every night*. (M20)

It was noticed in the mothers’ statements, in general, that protocols, manuals, flowcharts, booklets and other guidance tools are relevant and should be considered for support purposes. These, however, need to be conducted based on approaches that favor mothers’ autonomy, in order to empower and equip them for daily learning and to attribute meaning to different day-to-day experiences.

### Dealing with the certain and the uncertain

It was noted, in the speeches of several participants, that the excessive amount of parallel information leads to doubts and ends up generating uncertainty about what is right or wrong. In the speech of one participant, in particular, it was evident that the mother knows and recognizes what is best for her child and “feels what is right for her baby”. Furthermore, the mother needs to be equipped to put parallel opinions into perspective and to live with everyday adversities.

*He cried a lot, he didn’t know if it was colic or not, everyone said different things. The pediatrician said it was normal, but I didn’t feel safe. Then I cried a lot too.* (M5)

*One told me this and the other that. Sometimes I didn’t know who to believe and what to consider as right or wrong. As mothers, we need to feel what is right for our baby.* (M17)

Even though the participants have sought information throughout prenatal care and have relied on professionals specialized in the area, the feeling of caring for a newborn at home involves, to a large extent, a unique journey that demands expanded analysis and decision-making. continuous decisions.

*As a first-time mother, I was afraid that she wasn’t latching on correctly. The whole time I had to analyze whether the milk was enough, among millions of other decisions.* (M14)

*I felt very insecure. I could have prepared myself better to know how to deal and make decisions at each moment.* (M19)

*My husband worked in another city all day. So I was literally alone with the baby. I did everything alone. I never knew if I was making the right decision. I had a lot of insecurity and everything you can imagine.* (M21)

The feeling of insecurity and the inability to deal with the notions of right and wrong generated, in most participants, enormous discomfort and emotional exhaustion. Because it was a unique journey, each mother was responsible for her decisions, which were guided by existential experiences and learning that expanded in interactivity and the mother-child bond.

### Support network in the (de)organizing process

It was noticed, in some statements, that several participants had a support network, but many others did not. And, even for those who relied on this network, the process of reorganizing the daily care routine for the newborn required unique adaptations and learning. One mother, in particular, mentioned that she had no one to talk to and share her doubts and anxieties.

*I thought I was organized and had a network, but at the time I saw that it wasn’t quite like that. I had to reorganize myself in the middle of the postpartum period, learn new things and seek more support.* (M12)

*In my experience and vision, I think if I had my family around it would have been very different. It would have been calmer, not only because of having someone to help with the baby’s care, but also to talk, share experiences, give advice. I had no one to talk to and that was really bad.* (M18)

The support network was characterized in addition to the number of people involved and committed to caring for the newborn. In this support network, the possibility of creating a space for listening, dialogue and exchange of experiences is considered. In this sense, it was noticed that in addition to information, suggestions and regulations, mothers want to be welcomed, heard and supported in their decisions. Some participants also emphasized the importance of consultancy and advice provided by health professionals, which enable greater psychological preparation and safety.

*The support network is important when it creates spaces for dialogue and exchange of experiences. This comforts and facilitates overcoming difficulties in this period of adaptation.* (P16)

*I have a support network that helps me. My husband works all day, but when he comes home he helps me. I also have my mother-in-law and my sisters-in-law.* (M13)

*I consulted with an obstetric nurse. She helped me a lot with breastfeeding. I didn’t have one during my first pregnancy, but now I’ve learned a lot, especially in relation to the psychological preparation that breastfeeding requires.* (M9)

Although prenatal consultations were mentioned as relevant, one participant, in particular, highlighted the urgency of clear and safe professional information at the time of hospital discharge. The same participant also referred to the importance of home visits in the newborn’s first 28 days, conducted by qualified professionals.

*I consider it important to provide guidance when leaving the hospital. Information about baby care, sucking, latching, handling these things. Another thing that I think is very important is to have weekly monitoring by a professional, for up to 28 days, to check the condition of the mother and child.* (M8)

The support network, according to the participants, is made up of different family members (husband, mother-in-law, sisters-in-law and others), who share the same anguish and learning. In addition to the support network, it is necessary to encourage spaces for reception, dialogue and exchange of experiences, in which the mother feels supported and encouraged in her daily decisions. In this sense, it is important to design specific health support policies for mothers and other family members.

## DISCUSSION

Important progress is evident in terms of qualification of maternal and child health, although the reduction in maternal and neonatal mortality rates remains slow in Brazil. Studies^(17,18,19)^ demonstrate that the first 28 days of the newborn, in a home environment, deserve special attention. The focus, however, cannot only be on the newborn, but also on the mother who, equally, demands differentiated attention, especially on first-time mothers, as expressed by one of the participants: “I want them to listen to me and Don’t tell me what I should do”.

The expression “I want them to listen to me and not tell me what I should do” denotes an interventionist path that is still strongly guided by protocols and punctual and linear information, which does not consider the uniqueness of the mother caring for a newborn in an environment home. From this perspective, complexity thinking induces thinking that expands, contextualizes and singularizes professional practices, in order to understand the part in the whole, as well as the whole in each singular part^(14,15)^.

The excessive number of parallel protocol information was pointed out by study participants as a divisive practice that generates insecurity and doubts about what is right or wrong. In this sense, the author of complexity thinking emphasizes that it is urgent to rethink reform and reform thinking towards a well-rounded head, in order to contribute to the development of thinking autonomy. It supports the flexible organization of pertinent information and knowledge inserted in its global context, without however losing sight of its singular and multidimensional character^(14)^.

Mothers, in general, recognize the relevance of the information, but wish to be welcomed in their needs and provided with guidance in relation to the care of the newborn without impositions and previously established standards. They present specific demands related to possible complications that the newborn may present. Although the needs of caring mothers are similar to those of caring fathers, they express them in a more enlightened and open way, as fathers tend to hide their own concerns and have difficulties in talking about their feelings and asking for support^(20)^.

The relevance of the postpartum support network is, from this perspective, of fundamental importance for the (re)organization of daily life and new learning in the care of the newborn. Formal social support is desired in the postpartum period, especially among first-time mothers. Given the lack of distinction between formal social support and informal support in previous studies, it is necessary to expand research to better understand the specificities of formal social support for mothers during this particularly vulnerable period^(21)^. This clarity of evidence can help health professionals to better support the woman/mother in the postpartum journey and in the care of the newborn.

More than caring for the newborn, the focus of most studies, the caring mother needs a strengthened social support network (formal or informal) so that she can be welcomed, listened to and supported in her decisions. In addition to parallel information, mothers want to be heard about the experience of being a mother of a child who is unique to them and who, equally, demands unique care, which is not found in books. The first days postpartum constitute, in a woman’s life, a (re)organizing period considering that she needs to deal with the demands of her newborn baby, with her own care needs and, also, adapt to physiological and psychological changes^(22,23)^.

Studies show, from this same perspective, that a significant number of women suffer psychological disorders due to their perceived inability to care for their newborn both in a hospital and at home. Support from family caregivers can expand and strengthen a woman’s self-efficacy, confirm her role and identity as a mother and, in this way, improve her health and well-being. Home visit services in the first weeks of postpartum, through continued care from a qualified team, are also an important strategy for improving maternal and child health^(24,25)^.

The care of newborns in a home environment, especially in cases where first-time mothers are the main caregivers, needs to be explored in the light of references that expand theoretical possibilities and intervention approaches. Newborn care needs to transcend linear and normative perspectives, generally induced by pre-established protocols, booklets and resources. In addition to specific information, care must consider the unique dimension of each newborn, without disregarding the mother’s care needs, who, in turn, demand equally unique and complex care.

The thought of complexity evokes, in this sense, the multidimensional character of reality, uncertainty, disorder or absolute order, among other attributes. Under this impulse, a new approach is conceived in which the complexity of thought transcends the determinism of punctual and linear practices, which tend towards new reductionism. Complexity thinking does not claim to be superior to other knowledge, but presents itself as capable of aggregating previous knowledge and contextualizing it without reducing it to the blindness of the simplifying paradigm^(26)^. This thinking makes it possible, in this direction, to enhance interactions and consider the experience of each woman/mother as a distinct and plural adaptive process, which must be welcomed and understood by health professionals who work within the family context.

Human beings have the potential to think and act on their own in different existential realities and, from situations of disorder, reach the most advanced order/organization, in this case of caring for newborns in a home environment. Complexity thinking induces self-organization on the part of the person involved (caregiving mother), based on their own experiences, learning, routines and daily (re)adaptations. Therefore, the process of caring for a newborn in a home environment can only be understood in the light of a thought that is complex and whose understanding requires acceptance of the multiple dimensions or threads that are woven together to give meaning to the lived experience. The construction of singular and multidimensional care involves considering knowledge and practices that transcend the linearity of doing and that capture the values, beliefs, convictions and uniqueness of both mother and baby^(26,27)^.

Conceiving care for newborns goes back, from the perspective of complexity thinking, to a historical-hegemonic tradition marked by prescriptive, predictable and normative relationships, in which protocols were determined based on a subject-object relationship, without considering adaptive peculiarities of each woman/postpartum woman. In this relationship, the mother caregiver was deprived of autonomy and was subjugated to prescriptive and controlling knowledge, that is, the mother was deprived of autonomy, knowledge and life meanings related to the care of the newborn. In this simplifying logic, therefore, disorder, contradictions, uncertainties or any disordered element were disregarded^(2,26)^.

Advancing the quality of health care means, in the logic of complexity thinking, welcoming and apprehending the mother caregiver in her uniqueness and complementarity, as well as transcending linear perceptions and achieving an integrated, collaborative and inseparable dynamic between theoretical knowledge and lived knowledge^(28)^. This evolution, which does not exclude disorder, implies recognizing that the quality of care is associated with the ability to evolve in the inseparability and dialogicity between the notions of order and disorder, with a view to (re)organization. How, however, can we promote a compression of care, based on the concrete experiences and daily learning of mothers caring for newborns in a home environment?

Under this instigating and prospective impulse, it is advisable to consider the (re)organizing dimension of the father, who shares the pregnancy-puerperal journey. In this study, however, the father was not considered the object of investigation. However, the relevance of complementary, competing, antagonistic and dialogical phenomena is highlighted as progress, which evokes the necessary multidimensionality and the apprehension of the part in the whole as well as the whole in the part, based on a logic that is neither reductive nor totalizing, but reflective, dialogical and evolutionary^(29)^.

Newborn care in a home environment must be ensured by qualified caregivers, adequate environmental conditions and technologies that induce best practices, similar to the Kangaroo Mother Care used in neonatal and pediatric units. Caring for newborns at home requires differentiated attention and incessant support to overcome daily challenges and promote a constructive and favorable (re)organization. This path, supported by interactive technologies, expands the family’s bond in care, favors skin-to-skin contact, and intensifies the mother-child bond, among other benefits^(30)^.

Thinking about and understanding the best practices in home care services implies, in short, evolving in thought and action, the perspective of which is within the reach of any human being and professional area. Personal and family (re)adaptation and (re) organization are complex phenomena, but they are within the reach of all mothers, fathers and families, as long as each one is willing to evolve in thought, reflection, understanding, collaboration and dialogue between notions of order and disorder, certain and uncertain, simple and complex.

### Study limitations

A limitation of this study is the impossibility of carrying out all interviews in person, given the geographical distance of some participants. Another limitation is associated with the non-inclusion of the father in the investigative process, considering that he goes through a process of adaptation and (re)organization similar to that of the mother caring for a newborn in a home environment.

### Contributions to the area of Nursing and Health

The contributions of this study to the advancement of Nursing and Health science are associated with the perception that the mother caring for a newborn in a home environment requires differentiated attention, complemented by a formal or informal social support network. Although several studies have already been carried out on this topic, the study in focus aims to broaden perspectives and uncover theoretical possibilities, in light of complexity thinking, that consider the mother and the newborn in their singular and multidimensional dimension.

## FINAL CONSIDERATIONS

The care of newborns in a home environment, in the perception of mothers, requires differentiated attention and a formal or informal support network that considers the unique specificities of each woman/mother in the personal, family and social spheres. Therefore, in addition to the social support network, it is important to rethink home intervention approaches.

The perception of mothers who cared for newborns at home suggests the expansion of spaces for reception, dialogue and exchange of experiences so that they feel supported and encouraged in their daily learning. In addition to normative instructional technologies, mothers want to be heard and understood in their needs and supported in their decision-making.

The present study reinforces the relevance of inducing complementary, competing, antagonistic and dialogic processes, which evoke the necessary multidimensionality and the apprehension of the mother/father-baby binomial, based on a logic that is neither reductive nor totalizing, but reflective and evolutionary. Therefore, new studies are suggested that consider the pregnancy-puerperal journey from the perspective of complexity thinking.
